# Supplementary material for: WordCommentsAnalyzer: A windows software tool for qualitative research
Source: F1000Res. 2018 Sep 4;7:536. Originally published 2018 May 3. [Version 2] doi: 10.12688/f1000research.14819.2 (PMC6137411; doi:10.12688/f1000research.14819.2)
Supplement: Supplementary file 3 [file f1000research-7-17614-s0002.tgz › b2a79496-4ada-47c4-9682-a99ce867836d.zip!/Eight Iranian Health Journals/Journal of Health System Research _48.docx]

© Journal of Health System Research . This work is licensed under CC BY-NC 4.0 (https://creativecommons.org/licenses/by-nc/4.0/).

Volume & Issue: ویژه نامه بهداشت محیط؛ 1389

Title: مروری بر نیترات در آب آشامیدنی و اثرات بهداشتی آن با تأکید بر اثرات سرطان‌زایی آن در انسان

http://hsr.mui.ac.ir/index.php/jhsr/article/view/70

در طی دو قرن اخیر، میزان تولید و مصرف نیترات به خصوص در بخش کشاورزی افزایش چشمگیری داشته است. در حال حاضر بسیاری از نقاط جهان حتی در ایران با مشکل بالا بودن غلظت نیترات در آب آشامیدنی روبه‌رو هستند که مهم‌ترین علت آن ورود رواناب‌های کشاورزی و فاضلاب‌های شهری و صنعتی به منابع آبی به خصوص آب‌های زیرزمینی می‌باشد. مصرف آب آلوده به نیترات، همراه با مواد غذایی محتوی نیترات بالا می‌تواند باعث ورود مقدار زیادی نیترات به بدن گردد. از دیر باز ثابت شده که غلظت بالای نیترات در آب باعث بیماری متهموگلوبینمیا در کودکان می‌شود و حتی استانداردهای نیترات در آب آشامیدنی بر مبنای حفاظت کودکان در برابر این بیماری وضع شدند. اما مسأله‌ای مهم که در چند دهه اخیر بین محققان مورد بحث می‌باشد، پتانسیل سرطان‌زایی نیترات در آب آشامیدنی می‌باشد، به طوری که بسیاری از مطالعات شیوع انواع سرطان‌ها مانند سرطان معده، تیروئید و مثانه را با سطح نیترات آب آشامیدنی ارتباط دادند. با این حال برخی مطالعات این رابطه را تأیید نکردند. در این نوشته به بررسی نتایج مطالعات و تحقیقات انجام گرفته در سطح جهان در خصوص ارتباط بین نیترات آب آشامیدنی و خاصیت سرطان‌زایی آن پرداخته می‌شود. همچنین در مورد دیگر مسایل مرتبط با نیترات از جمله رژیم غذایی محتوی نیترات، متابولیسم نیترات در بدن انسان، دیگر اثرات مهم مرتبط با نیترات یعنی بیماری متهموگلوبینمیا و اختلالات دوران جنینی پرداخته می‌شود. در نهایت رویکرد کلی در مورد کنترل نیترات در آب آشامیدنی ارایه می‌گردد.

Title: بررسی تأثیر روان‌آب‌ها و پساب تصفیه‌خانه فاضلاب بر پارامترهای کیفی آب رودخانه زاینده رود

http://hsr.mui.ac.ir/index.php/jhsr/article/view/91

مقدمه: کیفیت آب رودخانه‌ها به عوامل مختلفی از جمله عوامل هیدرولوژیکی، فیزیکوشیمیایی و بیولوژیکی وابسته است. رودخانه‌ها از لحاظ توسعه اجتماعی، اقتصادی و سیاسی بسیارحائز اهمیت هستند. رودخانه زاینده ‌رود تنها رودخانه حوضه مرکزی ایران است که دارای آب شیرین دائمی می‌باشد. این رودخانه اهمیت بسیاری در تأمین آب آشامیدنی، حفظ حیات صنعتی استان و تامین آب کشاورزی دارد. بنابراین مدیریت و کنترل کیفیت این منبع آبی از اهمیت ویژه‌ای برخوردار است. در این مطالعه تأثیر روان‌آب‌های شهر اصفهان و پساب تصفیه‌خانه فاضلاب بر برخی پارامترهای کیفی آب رودخانه زاینده ‌رود مورد بررسی قرار گرفته شده است. روش‌ها: نمونه‌برداری جهت انجام بررسی‌های آزمایشگاهی از پنج ایستگاه‌ در طول رودخانه در حد فاصل ایستگاه هیدرومتری موسیان تا ورزنه انجام شد. پارامترهای اندازه‌گیری شده در این مطالعه شامل تعیین میزان نیتروژن نیتراتی، فسفات، آمونیوم، BOD5، COD و pH می‌باشد. در هنگام نمونه‌برداری اشل رودخانه در سه ایستگاه موسیان، چوم و ورزنه که دارای ایستگاه هیدرومتری بود نیز قرائت گردید. یافته‌ها: دبی رودخانه زاینده رود در اسفند ماه حداقل و در فروردین ماه حداکثر می‌باشد. مقدار فسفات بر حسب فسفر در تمام ایستگاه‌ها و در تمام طول دوره مطالعه از 049/0 تا 7/2 متغیر بوده است. تغییرات pH در آب رودخانه در چهار ایستگاه بین 2/7 تا 6/8 در هنگام نمونه‌برداری متغیر است. نتیجه‌گیری: آب رودخانه زاینده رود حتی در شرایطی که بارندگی صورت نمی‌گیرد از روان‌آب‌های اصفهان متأثر می‌گردد. اما شدت تأثیر پساب تخلیه شده از تصفیه‌خانه فاضلاب جنوب اصفهان بسیار زیادتر می‌باشد.

Title: بررسی و مقايسه تجمع فلزات سنگين در آب و خاك و گياه تالاب بين‌المللي گاوخوني در سال‌های 85-1381

http://hsr.mui.ac.ir/index.php/jhsr/article/view/142

مقدمه: تالاب گاوخونی یکی از مهم‌ترین تالاب‌های ایران است. در سال‌های اخیر به دلیل انجام فعالیت‌های صنعتی و کشاورزی در حوزه‌ی آبخیز تالاب، کیفیت محیط زیست آن تحت تأثیر قرار گرفته است. این تحقیق به منظور بررسی تعدادی از پارامترهای شیمیایی و غلظت فلزات سنگین در آب و خاک و گیاه تالاب گاوخونی و مقایسه نتایج با استانداردها و همچنین نتایج حاصل از بررسی‌های انجام شده در سال‌های گذشته، از طریق آنالیز نمونه‌های برداشت شده از محیط تالاب در اردیبهشت سال 1385 انجام گرفت. روش‌ها: نمونه‌های آب پس از انتقال به آزمایشگاه جهت تعیین فلزات سنگین، pH، هدایت الکتریکی و کلراید مورد آنالیز قرار گرفت. نمونه‌های خاک برداشت شده از سطح و عمق 10 سانتی‌متری به همراه نمونه‌های گیاهی بعد از خشک شدن در حرارت 103 تا 105 درجه سانتی گراد در مجاورت اسید سولفوریک و هیدروژن پراکسید هضم شدند (9). این نمونه‌ها پس از هضم و عبور از فیلتر تا حجم معینی رقیق شدند و سپس جهت تعیین فلزات سنگین توسط دستگاه جذب اتمی بر اساس روش A 303 استاندارد متد (7) مورد آنالیز قرار گرفتند. یافته‌ها: نتایج نشان داد که میانگین غلظت فلزات Cd، Cr، Cu، Mn، Ni، Pb و Zn در آب نقاط تعیین شده از پل ورزنه تا کوه سیاه تالاب به ترتیب برابر 73/0، 054/0، 76/0، 058/0، 48/0، 14/1 و 075/0 میلی‌گرم بر لیتر می‌باشد که این مقادیر از استانداردهای مربوط به آب آشامیدنی و آب مناسب برای کشاورزی نیز بیشتر است. همچنین غلظت‌های به دست آمده برای فلزات مذکور در خاک و گیاه از حدود استاندارد مربوط بیشتر می‌باشد. مقادیر مربوط به هدایت الکتریکی و کلراید آب تالاب نیز بیانگر آلودگی شدید آن می‌باشند که در صورت تداوم فعالیت‌های پیش گفته و تجمع آلاینده‌ها، موجودیت تالاب تحت تأثیر و تخریب قرار خواهد گرفت. نتیجه‌‌گیری: مقایسه نتایج حاصل از بررسی‌های انجام شده در سال‌های1381 و 1383، بیانگر روند افزایشی غلظت آلاینده‌های مذکور در آب و خاک تالاب می‌باشد، در حالی که مقایسه نتایج حاصل از این تحقیق و بررسی‌های فوق بیانگر وجود تغییراتی مبنی بر کاهش بعضی از پارامترها در آب و خاک تالاب نسبت به سال‌های گذشته است. دلیل این امر بارش مناسب و پر آب شدن تالاب در سال 1385 می‌باشد.

Title: بررسی کمیت و کیفیت مواد زائد جامد صنعتی (مطالعه موردی: استان چهارمحال و بختیاری در سال 1388)

http://hsr.mui.ac.ir/index.php/jhsr/article/view/109

مقدمه: از نظر اقتصادی و زیست‌محیطی، هدف اصلی در مدیریت زائدات صنعتی کاهش تولید این دسته از مواد است که با توسعه استراتژی‌های استفاده مجدد، بازیافت ضایعات و همچنین ارتقای توان بهره‌گیری از قوانین و تکنولوژی‌ها در جهت عدم تولید زائدات می‌توان به این هدف دست یافت. بررسی دقیق کمی و کیفی زائدات که در بخشی از صنعت تولید می‌شود، مبنای مدیریت صحیح مواد زائد صنعتی است. بنابراین با توجه به گسترش رو به رشد صنعت، نیاز به تدوین روش‌های کارآمد و متناسب با شرایط منطقه تصمیم گرفته شد که اطلاعات پایه و فنی مورد نیاز جمع‌آوری، اندازه‌گیری و مورد تحلیل قرار گیرد. روش‌ها: در این مطالعه، با توجه به پراکندگی، نوع و اندازه واحدهای صنعتی موجود در استان چهارمحال و بختیاری، پسماندهای تولید شده به وسیله تعداد 309 واحد صنعتی طی مدت 8 ماه از نظر کمی و کیفی مورد بررسی و اندازه‌گیری قرار گرفت. جمع‌آوری اطلاعات از طریق بررسی‌های میدانی و استفاده از روش‌های اندازه‌گیری صورت گرفت. یافته‌ها: بررسی‌های این مطالعه نشان می‌دهند که میزان کل زائدات تولید ‌شده در شهرک‌های صنعتی مورد مطالعه، 1246 تن در ماه بود که 2/48 درصد در صنایع غذایی، 9/14 درصد در صنایع فلزی، 7/6 درصد در صنایع شیمیایی، 22 درصد در صنایع کانی غیر فلزی، 9/0 درصد آن در صنایع نساجی، 6/0 درصد در صنایع برق و الکترونیک و 5/6 درصد آن در صنایع سلولزی تولید می‌شوند. نتایج نشان می‌دهند که نزدیک به نیمی از کل پسماندهای صنعتی تولیدی از محدوده مطالعه، موادی هستند که قابل تجزیه بیولوژیکی می‌باشند و تخلیه آن‌ها بدون رعایت ضوابط زیست‌محیطی منجر به آلودگی کوتاه مدت و ایجاد شرایط مزاحمت‌زا در محیط پذیرنده می‌شوند. همچنین قسمت‌های دیگر پسماندهای بررسی شده دارای کیفیتی است که بازیافت آن‌ها به چرخه مصرف از ارزش اقتصادی قابل توجهی برخوردار است و در صورت انتقال به محیط زیست منجر به آلودگی‌های بلند مدت و خطرناک خواهند شد. نتیجه‌گیری: با توجه به استقرار نامناسب بسیاری از صنایع از نقطه‌نظر جغرافیایی، خطرات بهداشتی و زیست‌محیطی آن متوجه جوامع و آب‌هاي سطحي می‌شود. بنابراین ضروری است که در مكان‌يابي صنایع به ویژه آن دسته از واحدهای صنعتی که داراي پسماندهاي ويژه می‌باشند، دقت بیشتری صورت گیرد و با اعمال و اجرای مقررات سخت‌گیرانه در برابر صنایع موجود، گامی مؤثر در کاهش اثرات تخریبی آن‌ها برداشته شود.

Title: بررسی حذف فنل با استفاده از زئوليت اصلاح‌شده به وسیله كلريد آهن از محلول‌های مایی

http://hsr.mui.ac.ir/index.php/jhsr/article/view/112

مقدمه: صنایع متعدی مثل پالایشگاه‌های نفت ‌خام، کارخانه‌های پتروشیمی، فراوری روغن زیتون، سازندگان حشره‌کش‌ها و فعالیت‌های نفتی، فاضلاب مقادیر قابل توجهی فنل و مشتقات آن را تولید می‌نمایند. به دلیل این که فنل به عنوان ترکیب خطرناک سمی و پراهمیت شناخته شده است، باید قبل از تخلیه جریان‌های آلوده به محیط ‌زیست، تصقیه شوند. این مطالعه با هدف کلی بررسی امکان‌سنجی استفاده از زئولیت (کلینوپتیلولایت) اصلاح‌شده با کلرید آهن به عنوان یک جاذب در حذف فنل از فاضلاب سنتتیک انجام شد.  روش‌ها: پس از تهيه زئولیت، ابتدا نمونه جمع‌آوری شد، خرد شد و با استفاده از الک‌های استاندارد ASTM با اندازه مش20 (85/0 ميلي‌متر) دانه‌بندی گردید. زئولیت دانه‌بندی شده با کلرید آهن اصلاح گردید. جهت تعیین ترکیب شیمیایی زئولیت اصلاح‌شده و سطح ویژه آن از تکنیک‌های پراکنش پرتو ایکس و ایزوترم BET با نرم‌افزار بیلوسرپ استفاده شد. در این مطالعه، پارامترهایpH  (3، 7 و 12)، غلظت اولیه فنل (25 تا 200 میلی‌گرم بر لیتر)، زمان تماس (20 تا 240 دقیقه) و دوز جاذب (25/0–3 گرم در 250 سی‌‌سی) در یک راکتور ناپیوسته مورد بررسی قرار گرفت. غلظت فنل در نمونه‌های مختلف با استفاده از اسپکتروفتومتر در طول موج 500 نانومتر اندازه‌گیری شد. یافته‌ها: نتایج بررسی‌های جذب نشان می‌دهند که میزان جذب/حذف فنل با افزایش غلظت اولیه فنل، دوز جاذب و با افزایش pH (3 تا 12) افزایش می‌یابد. جذب فنل بعد از مدت 100 دقیقه به تعادل می‌رسد و pH بهینه در جذب فنل با زئولیت اصلاح شده 3 می‌باشد. داده‌های جذب، از مدل ایزوترم جذب لانگمیر (98/0 = R2) تبعیت می‌کنند. نتیجه‌گیری: نتایج این مطالعه نشان می‌دهند که زئولیت اصلاح‌شده قابلیت خوبی در حذف فنل دارد. جذب فنل در pH‌های پایین بیشتر و با افزایش pH میزان جذب کاهش می‌یابد. با توجه به ارزان بودن زئولیت و سادگی اصلاح آن، این جاذب را می‌توان در حذف آلاینده‌های خطرناک در آب و فاضلاب به کار گرفت.

Title: بررسي امکان افزايش کارايي عملکرد سيستم بدون تماس پرتوتابي فرابنفش در گندزدايي آب آشاميدني

http://hsr.mui.ac.ir/index.php/jhsr/article/view/115

مقدمه: سيستم‌هاي بدون تماس پرتوتابي فرابنفش متداول، با وجود سادگي و ارزان بودن، کارايي کمي در گندزدايي آب دارند. هدف اين مطالعه، بررسي امکان افزايش کارايي اين سيستم‌ها با ايجاد اصلاحاتي در آن جهت افزايش ميزان تماس آب با پرتوي فرابنفش و در نتيجه گندزدايي مؤثر مي‌باشد. روش‌ها: در اين مطالعه دو مدل ارتقا يافته با مدل متداول پرتوتابي بدون تماس فرابنفش مقايسه شد. مدل آبشاري بر اساس پرتودهي لايه‌هاي آبشاري آب در دو طرف لامپ بود که در نتيجه از تمام پرتوي منتشره از لامپ فرابنفش استفاده شد. در مدل پلکاني نيز لامپ در بالاي پله‌هاي فلزي براق قرار گرفت. نتايج حاصل از گندزدايي آب در دو دبي 2/0 و 4/0 ليتر در ثانيه در اين دو مدل، در شرايط مشابه با مدل متداول مقايسه شد. کارايي گندزدايي در اين مطالعه، توسط حذف کليفرم‌هاي مدفوعي موجود در آب اندازه‌گيري شد که در کدورت‌هاي NTU 5/0، 10، 20 بررسي شدند. يافته‌ها: راندمان گندزدايي در کدورت آب NTU 5/0، در مدل آبشاري با 65/3 لگاريتم کاهش کليفرم‌هاي مدفوعي بيشتر از مدل متداول با 93/2 لگاريتم کاهش بود (05/0 > P). همچنين اين مدل براي گندزدايي آب‌هاي با کدورت تا NTU 20 نيز مناسب بود. با این وجود مدل پلکاني کارايي کمتري نسبت به مدل متداول داشت. نتيجه‌گيري: ميزان راندمان گندزدايي در مدل آبشاري در مقايسه با مدل متداول در مقادير بالاتر جريان آب و کدورت بيشتر، کاهش چنداني نيافت. بنابراين، استفاده از اين مدل در مقياس‌هاي بزرگتر و براي تصفيه‌خانه‌هاي کوچک آب توصيه مي‌شود.

Title: بررسی آلودگی میکروبی بیمارستان امام صادق (ع) شهرستان دلیجان در سال 1389

http://hsr.mui.ac.ir/index.php/jhsr/article/view/116

مقدمه: کنترل آلودگی‌های میکروبی یکی از مهم‌ترین راه‌های پیشگیری از انتشار بیماری‌های عفونی در امکان عمومی به خصوص در بیمارستان‌ها می‌باشد. در این مطالعه، هدف بررسی وجود انواع استافیلیکوک، باکتری‌های هتروتروفیک و نهایتاً 71 نوع از میکروارگانیسم‌های خانواده آنتروباکتریاسه همچون اشرشیاکلی و کلبسیلا می‌باشد که به عنوان شاخص‌هایی از تنوع میکروبی در هوا و سطوح موجود در بیمارستان از جمله درها و دیوارهای بخش‌های مختلف بیمارستان می‌باشند. همچنین بررسی امکان انتقال میکروب‌ها از طریق سیستم تهویه (هواساز) نیز از دیگر اهداف این مطالعه می‌باشد. روش‌ها: بررسی میکروبی در این مطالعه به دو بخش بررسی کیفیت میکروبی هوا و کیفیت میکروبی سطوح مختلف موجود در بیمارستان تقسیم‌بندی می‌گردد. برای بررسی هوا با کمک یک مکنده قوی (پمپ وکیوم)، هوا را از روی یک صافی میلی پور با قطر سوراخ‌های کمتر از 3/0 میکرون عبور داده شد تا میکروب‌های موجود در هوا پس از تغلیظ بر روی این صافی اندازه‌گیری شوند. جهت بررسی سطوح نیز با کمک ابزار مناسب (سوآپ) از سطوح نمونه‌برداری و به محیط کشت انتقال داده شد. در این مطالعه از کشت بشقابی برای شمارش باکتری‌های هتروتروفیک استفاده گردید. همچنین دو شاخص اشرشیاکلی و کلبسیلا نیز با کمک روش کشت تخمیر چند لوله‌ای مورد بررسی قرار گرفتند. برای شناسایی خانواده آنتروباکتریاسه نیز از کیت آزمون ای استفاده گردید. یافته‌ها: در میان شاخص‌های مختلف انتخاب شده، هوای بیمارستان عاری از اشرشیاکلی بود. میکروارگانیسم‌های موجود در هوای بیمارستان نیز شامل استافیلوکوکوس شلی فری، استافیلوکوکوس هایکوز و کلابسیلا پنومونیه بودند. همچنین شمارش باکتری‌های هتروتروفیک نشان می‌دهند که تعداد آن‌ها در هوای بیمارستان فراوان است. نتیجه‌گیری: با توجه به سنجش میکروب‌ها قبل و بعد از (فیلترهای) سیستم تهویه هوا (هواساز) مشخص شد، این سیستم (فیلتر‌های هواساز موجود در بیمارستان) کارایی کافی در حذف میکروارگانیسم‌ها را ندارد. بررسی سطوح همچون درها، دیوارها و ... وجود شاخص‌های ذکر شده در بیمارستان را نشان می‌دهد.

Title: بررسی میزان غلظت نیترات و نیتریت در چاه‌های آب شرب روستاهای اطراف شهرک صنعتی شهر ارومیه

http://hsr.mui.ac.ir/index.php/jhsr/article/view/37

مقدمه: ترکیبات نیترات و نیتریت از عوامل آلاینده آب‌های زیرزمینی محسوب می‌شوند، که در سال‌های اخیر غلظت آن‌ها در این منابع رو به افزایش می‌باشد. این تحقیق با هدف اندازه‌گیری غلظت نیترات و نیتریت در چاه‌های آب شرب روستاهای اطراف شهرک صنعتی ارومیه و مقایسه با استانداردهای مصوب انجام گرفت. روش‌ها: این مطالعه از نوع مقطعی- توصیفی- تحلیلی بود. نمونه‌برداری از 30 حلقه چاه به مدت یک سال در نقاط مختلف روستاهای اطراف شهرک صنعتی ارومیه انجام شد و غلظت نیترات و نیتریت، مورد اندازه‌گیری قرار گرفت. بر اساس نتایج حاصل از آزمایشات، میانگین سالیانه غلظت نیترات بر حسب -NO3 و نیتریت بر حسب -NO2 به ترتیب mg/L 46/17 و mg/L 008/0 به دست آمد، که بیشترین غلظت نیترات مربوط به فصل زمستان بود. برای مقایسه میانگین غلظت نیترات و نیتریت در فصول مختلف، از آزمون آنالیز واریانس یک طرفه استفاده گردید. یافته‌ها: این بررسی نشان داد که میانگین غلظت نیترات بین فصل بهار با تابستان و نیز بین فصل بهار با پاییز اختلاف معنی‌داری دارد (05/0 < P)، اما در میانگین غلظت نیتریت نمونه‌های آب در فصول مختلف اختلاف معنی‌داری مشاهده نگردید (05/0 < P). همچنین ارتباط معنی‌دار آماری بین مقادیر نیترات با عمق آب برقرار شد (76/0- = r، 001/0 < P) و با افزایش عمق آب، غلظت نیترات چاه‌ها کاهش یافت. نتیجه‌گیری: از یافته‌های این تحقیق می‌توان نتیجه گرفت که در حال حاضر کیفیت آب مورد مطالعه از نظر غلظت نیترات و نیتریت مطابق با استاندارد‌های آب شرب ایران می‌باشد؛ با این وجود کنترل مستمر این منابع آب ضروری است. از این رو پایش، تصفیه و دفع بهداشتی فاضلاب‌های تولیدی هم بایستی مورد توجه قرار گیرد.

Title: بررسی کیفیت شیمیایی و وضعیت کلرزنی آب آشامیدنی روستاهای شهرستان کاشان

http://hsr.mui.ac.ir/index.php/jhsr/article/view/36

مقدمه: طبق نظر سازمان بهداشت جهانی، آب مورد استفاده جهت مصرف انسان می‌بایست عاری از عوامل میکروبی و مواد شیمیایی خطر ساز برای سلامتی انسان باشد، بنابراین کنترل کیفیت آن از اهمیت خاصی برخوردار است. هدف این تحقیق تعیین کیفیت شیمیایی و وضعیت کلرزنی آب آشامیدنی در روستاهای شهرستان کاشان می‌باشد. روش‌ها: این مطالعه توصیفی- مقطعی در نیمه دوم سال 1386 بر روی آب آشامیدنی روستاهای شهرستان کاشان انجام گرفت. در این تحقیق پارامترهای فیزیکی و شیمیایی آب منابع تأمین آب، 1 مرتبه و کلر آزاد باقیمانده آب نقاط مصرف، 3 مرتبه مورد سنجش قرار گرفتند. یافته‌ها: نتایج این مطالعه نشان می‌دهند که غلظت نیترات، نیتریت، کلرور، کلسیم و مقدار pH در تمام روستاها در حد استاندارد می‌باشند. اما غلظت سولفات، منیزیم، سدیم، TDS و کدورت به ترتیب در 3/5، 3/5، 8/8، 3/5 و 5/3 درصد روستاها فراتر از استاندارد می‌باشد و غلظت فلوراید در 9/50 درصد روستاها پایین‌تر از دامنه مجاز است. همچنین به ترتیب 0/40، 5/1 و 8/34 درصد جمعیت به ترتیب روستاهای تحت پوشش، غیر تحت پوشش آب و فاضلاب روستایی و کل روستاها از آب با کیفیت مطلوب از نقطه نظر کلر آزاد باقیمانده استفاده می‌کنند. نتیجه گیری: براساس نتایج این مطالعه می‌توان گفت که متوسط غلظت اکثر عوامل شیمیایی در آب آشامیدنی روستاهای شهرستان کاشان در حد استاندارد 1053 ایران می‌باشند، اما غلظت فلوراید در بسیاری از روستاها کمتر از حد مجاز می‌باشد. همچنین شاخص مطلوبیت کلر باقیمانده آب آشامیدنی روستاهای شهرستان کاشان پایین‌تر از متوسط کشوری می‌باشد.

Title: بررسی کيفيت ميکروبی آب آشاميدنی روستاهای شهرستان کاشان و نقش شركت آب و فاضلاب روستايي در بهبود آن

http://hsr.mui.ac.ir/index.php/jhsr/article/view/45

مقدمه: هدف اين مطالعه تعيين کيفيت ميکروبی آب آشاميدنی روستاهای شهرستان کاشان و ميزان بهره‎مندی جمعيت اين روستاها از آب سالم از نقطه ‎نظر کيفيت ميکروبی در نيمه دوم سال 1386 مي‎باشد. همچنين نقش شركت آب و فاضلاب روستايي (آبفار) كاشان، در بهبود كيفيت ميكروبي آب آشاميدني مناطق روستايي اين شهرستان مورد بررسي قرار گرفت. روش‌ها: در اين مطالعه توصيفی- مقطعی، كيفيت ميكروبي آب تمامي 57 روستاي شهرستان، 3 مرتبه بر اساس 3 معيار كل كليفرم، كليفرم مدفوعی و شمارش بشقابي هتروتروفيك مورد سنجش قرار گرفت. يافته‌ها: در اين مطالعه ميانگين داده‌هاي حاصل از 3 مرحله نمونه‌برداري و آزمايش ارایه شده است. نتايج نشان می‎دهند که 100، 71/47 و 99/92 درصد جمعيت روستاهای تحت پوشش، غير تحت پوشش شركت آبفار و کل روستاهاي شهرستان كاشان از آب سالم از نظر کليفرم مدفوعی و همچنین 4/98، 2/21 و 88 درصد جمعيت روستاهای تحت پوشش، غير تحت ‌پوشش آب‌ و فاضلاب روستايی و کل روستاها از آب سالم از نظر کليفرم بهره‌مند بودند. بين كيفيت ميكروبي روستاهای تحت پوشش و غير تحت پوشش شرکت آبفار اختلاف معنی‎داری وجود دارد. نتيجه‎گيری: نتايج مطالعه حاضر بيانگر آن است که وضعيت روستاهای تحت‌ پوشش از لحاظ آلودگی مدفوعی بسيار عالی است، ولی برای روستاهای غير تحت ‌پوشش شرايطي نامناسبي حاكم است و به طور كلي كيفيت ميكروبي آب آشاميدني كل روستاها در حد شاخص ميکروبی كشور مي‌باشد. همچنين مشخص گرديد که نقش شرکت آب ‌و فاضلاب روستايی در تأمین آب سالم از نقطه نظر كيفيت ميكروبي، برای جمعيت روستايی بسيار مهم می‎باشد.

Title: مديريت مواد زائد جامد صنعتي توليدي در شهرک صنعتي بروجن

http://hsr.mui.ac.ir/index.php/jhsr/article/view/75

مقدمه: در كنار مسایل و موضوعات گوناگون محيط ‌زيست انساني، خطرات زيست‌محيطي ناشي از سوء مديريت مواد زائد صنعتي، يكي از مسایل اساسي كشور به حساب مي‌آيد. اين موضوع به ويژه در استان‌هایي از قبيل استان چهارمحال و بختياري كه در آن‌ها تمركز و تنوع منابع طبيعي وجود دارند و در چند سال اخير با رشد سريع تكنولوژي و صنعت روبرو هستند، از اهميت بيشتري برخوردار است. روش‌ها: جهت بررسي وضعيت دفع فعلي مواد زائد صنعتي در شهرك‌ صنعتي بروجن و بهسازي وضعيت موجود سيستم مديريت پسماند، در شهرک صنعتي بروجن از روش پرسش‌نامه استفاده گرديد كه به صورت حضوري از صاحبان و مسؤولين صنايع سؤال ‌گرديد و در پرسش‌نامه ياداشت شد و در نهايت از صنعت مورد نظر بازديد شد و شماتيك فرايند توليد، مشخص گردید. در اين پرسش‌نامه وضعيت بهره‌برداري، نوع فعاليت، نوع و مقدار مواد اوليه مصرفي، نوع و مقدار محصولات توليدي و زباله‌هاي توليدي توسط هر صنعت بررسي گرديد. يافته‌ها: در شهرک صنعتي بروجن به طور کلي 2200 کيلوگرم در روز زائدات بهداشتي در اثر فعاليت پرسنل در اين شهرک توليد مي‌شود. در بررسي‌هاي انجام شده، بر اساس ترکيب فيزيکي زباله، مشخص شد که با در نظر گرفتن تمام مواد قابل بازيافت، حدود 6/68 درصد وزن مواد زائد صنعتي در شهرک صنعتي بروجن کاهش خواهد يافت ولي تمام مواد قابل بازيافت را نمي‌توان بازيابي نمود. بنابراين کاهش وزن به حدود 61 درصد مي‌رسد. نتيجه‌گيري: بر اساس بررسي‌هاي ميداني جهت امكان احداث واحدهاي بازيافت در شهرك صنعتي بروجن، مي‌توان از تأسيسات بازيافت كاغذ در اين شهرك صنعتي استفاده نمود و با توجه به وجود صنايع بازيافت كاغذ در اين شهرك، پتانسيل خوبي در اين مورد وجود دارد. ايجاد تأسيسات بازيافت و پردازش پلاستيك مقرون به صرفه نيست. همچنين توصيه می‌شود كه زائدات آهني در يك نقطه مشخص در شهرك صنعتي جمع‌آوري شوند و پس از يك دوره مشخص به عنوان مواد اوليه به صنايع مادر فروخته شوند.

Title: بررسی کیفیت شیمیایی آب زیرزمینی منطقه سجاد شهرستان زرین‌‌‌شهر

http://hsr.mui.ac.ir/index.php/jhsr/article/view/50

مقدمه: منابع آب زیرزمینی به خصوص در مناطق خشك و نیمه خشك بسیار با اهمیت می‌باشند. فعالیت‌های بشر در زمینه‌های كشاورزی، صنعت و شهری پتانسیل بسیار بالایی برای آلوده نمودن این منابع داشته، باعث تقلیل کیفیت آن‌ها می‌گردد. تحقیق در زمینه كیفیت شیمیایی آب‌های زیر‌زمینی در تعیین عوامل آلاینده محیطی كه مرتبط با دفع نادرست فاضلاب‌های مختلف تولیدی هستند، بسیار حایز اهمیت است. در این تحقیق با اندازه‌گیری پارامترهای تعیین کیفیت آب و مقایسه آن با استاندارد، میزان تأثیر دفع فاضلاب صنعت فولاد، نشت آن به كانال زهكش منطقه و آب چاه‌های زیر‌زمینی منطقه سجاد شهرستان زرین‌شهر مورد بررسی قرار گرفت. روش‌ها: در این مطالعه تحلیلی- مشاهده‌ای كه به صورت مقطعی انجام شد، نمونه‌ها از 6 حلقه چاه به مدت سه ماه جمع‌آوری شدند و در مجاورت یخ به آزمایشگاه شیمی دانشكده بهداشت انتقال یافتند. پارامترهای EC، pH، COD، TDS و فلزات سنگین (سرب، كادمیم، كروم و نیكل) بر اساس روش‌های ارایه شده در کتاب استانداردهای آب و فاضلاب، مورد اندازه‌گیری قرار گرفت. جهت آنالیز داده‌ها از آزمون آماری t-test و از نرم‌افزار Excel جهت ترسیم نمودارها استفاده گردید. یافته‌ها: میزان پارامتر pH در آب چاه‌های مورد مطالعه در محدوده 3/8-6/6، میزان هدایت الكتریكی در محدوده 6388-3668 میكرو‌زیمنس بر سانتی‌متر و میانگین غلظت کل جامدات محلول برابر با 2619 میلی‌گرم در لیتر بود. میانگین غلظت COD و فلزات سنگین (كادمیم، سرب، كروم و نیكل) به ترتیب برابر با 22/30، 045/0، 062/0، 049/0 و 149/0 میلی‌گرم در لیتر به دست آمد. نتایج نشان داد كه به جز كروم میانگین غلظت سایر پارامترهای اشاره شده از استانداردهای آب شرب بالاتر می‌باشد. همچنین میزان آلودگی در چاه‌های شماره 1 و 4 از سایر چاه‌ها بیشتر بود. نتیجه‌گیری: بالا بودن غلظت پارامترها نسبت به میزان توصیه شده در استانداردها نشان می‌دهد كه آب چاه‌های منطقه مورد مطالعه در اثر تخلیه نادرست فاضلاب‌های صنعتی و كشاورزی آلوده شده است، بنابراین کنترل و تصفیه مناسب فاضلاب‌های تولیدی در منطقه و همچنین پایش آب‌های زیر‌زمینی ضروری می‌باشد.

Title: بررسی میزان بازدارندگی روغن‌های حاوی بی فنیل‌های چند کلره (PCBs) بر روی بیومس بی‌هوازی به روش آزمون فعالیت متان‌سازی ویژه (SMA)

http://hsr.mui.ac.ir/index.php/jhsr/article/view/52

مقدمه: در این مطالعه، میزان بازدارندگی غلظت‌های مختلف روغن حاوی آروکلرهای 1242 و 1254 PCBs (Polychlorinated Biphenyls) بر روی بیومس بی‌هوازی به روش آزمون فعالیت متان‌سازی ویژه (Specific Methanogenic Activity یا SMA) با استفاده از ویال‌هایی با حجم 120 میلی‌لیتر بررسی گردید. روش‌ها: نمونه‌های اصلی به دو صورت بدون استفاده از خاک آلوده به روغن حاوی PCBS و با استفاده از خاک آلوده به روغن حاوی PCBS و نمونه‌های شاهد در هر دو حالت فقط حاوی سوبستره و بدون افزودن روغن بودند. غلظت‌های مورد ارزیابی به سه دسته مقادیر کم، مقادیر متوسط و مقادیر بالا دسته‌بندی گردید و با یکدیگر و با نمونه‌های شاهد مقایسه شدند. یافته‌ها: یافته‌های حاصل از این مطالعه نشان داد که در ویال‌هایی که فقط حاوی 2 گرم خاک می‌باشند، 70 درصد حذف COD اتفاق افتاده است. همچنین سوبستره ورودی در شرایط بدون روغن حاوی PCB و در شرایطی كه روغن حاوی PCBs در مقادیر بین 02/0 میلی‌لیتر تا 3/0 میلی‌لیتر در آن وجود دارد، بدون تأخیر توسط میكروارگانیسم‌ها تجزیه می‌شود. نتیجه‌گیری: حداکثر متان‌سازی ویژه (CH4/g VSS. d mL 7/46) در ویال حاوی 1/0 میلی‌لیتر روغن اتفاق افتاد. بنابراین میزان غلظت بهینه روغن حاوی PCBs در ویال‌های SMA که حداکثر متان‌سازی ویژه را داشت، برابر 1/0 میلی‌لیتر به همراه 1/0 میلی‌لیتر سوبستره کمکی اسید استیک به دست آمد. در مقدار روغن ورودی 5/1 میلی‌لیتر به همراه سوبستره کمکی اسید استیک، غلظت بازدارنده بود.

Title: حذف کروم شش ظرفیتی از پساب شبیه‌سازی شده صنایع آبکاری توسط نانو ذرات مگهمایت

http://hsr.mui.ac.ir/index.php/jhsr/article/view/46

مقدمه: حذف فلزات سنگین از فاضلاب‌های صنعتی به وسيله نانو ذرات آهن در طي ساليان اخير بسيار مورد توجه قرار گرفته است. كروم به عنوان يكي ازآلاينده‌هاي دارای اهمیت در محیط زیست محسوب مي‌گردد و در غلظت‌های بالا در پساب صنایع آبکاری وجود دارد. نانو ذرات آهن مغناطیسی، با استفاده از مکانیسم‌های جذب سطحی، تعویض یونی و نیروهای الکترو‌استاتیک مي‌تواند در كنترل و حذف فلزات سنگین از فاضلاب‌های صنعتی مورد استفاده قرار گيرد هدف از انجام اين تحقيق بررسی کارآیی نانو ذرات مغناطیسی در حذف کروم شش ظرفیتی (VI) از پساب‌های شبیه‌سازی شده صنایع آبکاری و پارامترهاي مؤثر بر آن می‌باشد.  روش‌ها: نانو ذرات مگهمایت با استفاده از روش سل- ژل و با افزودن کلرور آهن دو و سه ظرفیتی در محیط مایی و تحت شرایط قلیایی تهیه گردید. سپس تأثیر عوامل مؤثر بر این فرآیند را که شامل غلظت نانو ذره، غلظت اولیه کروم، pH محلول، سرعت اختلاط و زمان تماس می‌باشند، بررسی گردید. یافته‌ها: یافته‌های این تحقيق نشان می‌دهد که در شرایط pH معادل 2، غلظت اوليه كروم ۱۰ ميلي‌گرم بر ليتر، نانو ذرات مگهمایت تولید شده با دوز 1 گرم بر لیتر، زمان تماس 5 دقیقه و سرعت همزن 250 دور در دقیقه حدود 86 درصد ازکرم (VI) حذف می‌گردد. خصوصیات نانو ذرات تولید شده شامل ساختار ذره، نوع ترکیب، اندازه و زتا پتانسیل آن‌ها نیز با استفاده از دستگاه‌های XRD، XRF و Zeta and particle sizer تعیین گردید. نتیجه‌گیری: از یافته‌های این پژوهش نتیجه‌گیری می‌شود که نانو ذرات تولیدی مگهمایت دارای کارایی بالایی درحذف کرم) VI) از پساب‌های شبیه‌سازی شده صنایع آبکاری بوده و راندمان حذف با pH رابطه عکس دارد.

Title: بررسی کارایی بیوراکتور غشایی (MBR) از نوع صفحه‌ای (Flat sheet) در تصفیه بیولوژیکی فاضلاب شهری تصفیه‌خانه جنوب اصفهان در مقیاس پایلوت

http://hsr.mui.ac.ir/index.php/jhsr/article/view/48

مقدمه: هدف از انجام این مطالعه بررسی کارایی بیوراکتور غشایی (MBR یا Membrane bioreactor) از نوع صفحه‌ای (Flat sheet) در حذف BOD5 و COD در تصفیه‌خانه فاضلاب شهری جنوب اصفهان بود. روش‌ها: در این مطالعه، یک بیوراکتور غشایی صفحه‌ای مستغرق با حجم 140 لیتر، با استفاده از غشای میکروفیلتراسیون (MF یا Microfiltration membrane) به مدت 109 روز و با زمان ماند هیدرولیکی 20 ساعت در تصفیه‌خانه فاضلاب شهری جنوب اصفهان تغذیه گردید. یافته‌ها: راهبری بیوراکتور در غلظت MLSS معادل 7800 میلی‌گرم در لیتر، به طور متوسط راندمان حذف COD و BOD5 به ترتیب میزان 95 درصد و 99 درصد را حاصل نمود. نتیجه‌گیری: سیستم FS-MBR (Flat sheet membrane bioreactor) می‌تواند به دلیل کیفیت بالای پساب خروجی و حذف BOD5 و COD طبق استاندارد‌های تخلیه به آب‌های پذیرنده در مقیاس بزرگ مورد استفاده قرار گیرد.

Title: حذف نيترات از آب با استفاده از جاذب گرانول هيدروكسيد فريك

http://hsr.mui.ac.ir/index.php/jhsr/article/view/72

مقدمه: يكي ازمنابع عمده آلودگي آب آشاميدني، نيترات است. اگر غلظت نیترات بالاتر از حد استاندارد (mgL-1 45 بر حسب نیترات) باشد؛ مصرف این آب سبب بروز بیماری متهموگلوبینمی در کودکان می‌شود. هدف از این مطالعه بررسی میزان جذب نیترات روی گرانول هیدروکسید فریک (GFH) می‌باشد. همچنین در این مطالعه، ميزان تأثير فاكتور‌هايي مانند غلظت اولیه نیترات، زمان تماس، جرم و اندازه ذرات جاذب در ظرفيت جذب تعيين شد. روش‌ها: این مطالعه بنيادي است و در مقیاس آزمایشگاهی انجام شد که در آن محلول‌هاي سینتيك نيترات در يك راكتور ناپيوسته در تماس با جاذب قرار داده شد تا سينتيك جذب، مدل شود. یافته‌ها: نتایج نشان می‌دهند که با افزايش غلظت اوليه نيترات، ظرفيت جذب در زمان كوتاه 2 تا 3 دقيقه به بيشترين حد مي‌رسد. ظرفيت جذب، با افزايش مقدار و كاهش اندازه ذرات جاذب كاهش می‌يابد. همچنين سينتيك جذب از الگوي واكنش‌هاي درجه دو كاذب پيروي مي‌كرد. نتیجه‌گیری: نیترات با این روش حذف، می‌تواند به زیر حد استاندارد (mgL-1 45 بر حسب نیترات) برسد. بهره‌برداری آسان و ظرفیت جذب بالای GFH، از مزایای به کارگیری این روش حذف است.

Title: تأثير آنتي‌بيوتيك‌ها بر فعاليت متان‌سازي ويژه بيومس بي‌هوازي

http://hsr.mui.ac.ir/index.php/jhsr/article/view/76

مقدمه: آنتي‌بيوتيك‌ها پس از تأثير بر بدن ميزبان، از بدن دفع شده و همراه ساير زائدات وارد فاضلاب‌ها مي‌شوند‌. آنتي‌بيوتيك‌ها مانع تصفيه بيولوژيكي فاضلاب‌ها شده كه خطراتي را در محيط زيست و به تبع آن براي سلامتي انسان‌ها به دنبال دارند. هدف از این پژوهش بررسی اثرات چند آنتي‌بيوتيك پر مصرف انسانی و دامی بر فرایند تصفيه بي‌هوازي فاضلاب می‌باشد. روش‌ها: به منظور ارزيابي ميزان بازدارندگي آنتي‌بيوتيك‌ها بر فعاليت متان‌سازي ويژه بيومس بي‌هوازي به روش Batch، درون ويال‌هاي 90ميلي‌ليتري، 15درصد حجمي بيومس و 70‌ميلي‌ليتر سوبستره ريخته شد و غلظت‌هاي مختلفي از آنتي‌بيوتيك‌هاي آموكسي‌سيلين، اكسي‌تتراسايكلين و تايلوزين به آن اضافه گرديد. دماي مطلوب 35‌درجه سانتي‌گراد در ويال‌ها توسط حمام آب گرم تأمين شد و برای اختلاط کامل محتویات ویال میکسر مغناطیسی استفاده شد. متان توليدي از طريق جايگزيني گاز با محلول KOH2‌ نرمال به عنوان جاذب ‌‌CO2 و برم تيمول بلو به عنوان انديكاتور اندازه‌گيري مي‌شد. مدت هر batch 10 روز طول مي‌كشید. آزمايش COD قبل و بعد از هر تست بر روي نمونه‌ها انجام مي‌گرفت. یافته‌ها: بر اساس یافته‌های حاصل از هر batch، اكسي‌تتراسايكلين، تايلوزين و آموكسي‌سيلين به ترتيب در غلظت‌هاي 8000، 9000و9000 ميلي‌گرم در ليتر بر فعاليت متان‌سازي بيومس بي‌هوازي كاملاً نقش باز‌دارندگی دارند. نتیجه‌گیری: نتایج این مطالعه نشان داد که با افزایش غلظت آنتی‌بیوتیک، حجم گازمتان توليدي به ازای واحد وزن بیومس كمتر مي‌شود.

Title: عملکرد سيستم زلال‌ساز اکسيلاتور در حذف کدورت

http://hsr.mui.ac.ir/index.php/jhsr/article/view/81

مقدمه: سیستم اکسیلاتور به دلیل داشتن مزایای زیادی از جمله تلفیق فرایند‌های اختلاط، لخته‌سازی و ته‌نشینی در یک واحد، امکان انجام همزمان سختی‌زدایی و زلال‌سازی و راندمان نسبتاً قابل قبول، امروزه مورد توجه طراحان تصفیه‌خانه آب می‌باشد که علی‌رغم به کار‌گیری آن در تعدادی از مناطق کشور، تحقیقات کافی در خصوص عملکرد این واحد در مقیاس واقعی و پایلوت انجام نشده‌است. روش‌ها: در این مطالعه تجربي به ‌منظور بررسی عملکرد سیستم اکسیلاتور در حذف کدورت با استفاده از منعقد‌کننده‌های شیمیایی (PACL و FeCl3)، سیستمی در مقیاس پایلوت طراحی و در شرایط کنترل شده (دمای 25 درجه سانتیگراد و  2/7 pH=) و شرایط متفاوت از نظر کدورت، دوز منعقد‌کننده و زمان ماند، بهره‌برداری شد‌. یافته‌ها: کارایی FeCl3در حذف کدورت در اکسیلاتور بیشتر از PACL بوده است. ضمن این که حداکثر کارایی سیستم اکسیلاتور در حذف کدورت، 95 درصد بوده که با تزریق 10 میلی‌گرم بر لیتراز ماده منعقدکننده FeCl3 پس از مدت زمان 240‌ دقیقه ته‌نشینی حاصل شده‌ است به ‌طوری‌ که کدورت NTU100 در آب خروجی به NTU5 کاهش یافته‌است. نتیجه‌گیری: نتایج نشان داد که تفاوت معنی‌داری بین زمان ماند و کدورت آب خروجی از پایلوت با ضریب همبستگی (98/0 -  r =) وجود دارد(05/0 p-value<).

Title: بررسی راندمان حذف سیلیکا آئروژل اصلاح شده در حذف فلزات سنگین از محلول‌های آبی

http://hsr.mui.ac.ir/index.php/jhsr/article/view/103

مقدمه: سیلیکا آئروژل، ذرات نانو متخلخل از سیلیکا بوده، سبک‌ترین ماده جامد‌ مصنوعی می‌باشد. از ویژگی‌های بارز این ماده، مساحت سطح بالا ( m2.g-11500)، دانسیته کم (g.cm-303/0 <) و میزان تخلخل بالا (99 درصد) می‌باشد. در این پژوهش، سیلیکا آئروژل به عنوان جاذب کاتیون‌های فلزات سنگین از محلول‌های آبی، مورد بررسی قرار گرفت. روش‌ها: سیلیکا آئروژل به روش سل ژل سنتز شد و سطح آن توسط آمینو‌ پروپیل تری اتوکسی سیلان اصلاح شد. سیلیکا آئروژل اصلاح سطح شده با روش‌های SEM و BET مطالعه شد. سپس، توانایی آن در جذب کاتیون‌هایPb2+، Cd2+، Zn2+ و Ni2+ بررسی شد. در نهایت پارامترهای مؤثر بر جذب از جمله pH برابر (12-2)، زمان تماس (48-0 ساعت)، میزان جاذب (5/1-0 گرم) و غلظت فلزات (500-0 میلی‌گرم بر لیتر) مورد بررسی قرار گرفت. یافته‌ها: جذب بهینه برای تمام کاتیون‌ها در pH برابر 6 و برای کادمیوم در pH برابر 8 رخ داد. زمان تماس بهینه نیز برای جذب تمام کاتیون‌های مورد مطالعه برابر ۲۴ ساعت بود. بررسی ایزوترم‌های Langmuir و Freundlich نشان داد که داده‌های به دست آمده از کاتیون‌های مورد بررسی با هر دو ایزوترم مطابقت دارد. بالاترین ظرفیت جذبی جاذب به ترتیب g 45/45، g 71/35، g 00/40 و g 48/34 از کاتیون‌های Pb2+، Cd2+، Ni2+ و Zn2+ بر mg جاذب به دست آمد. نتیجه‌گیری: به طور کلی نتایج حاصل از آزمایشات انجام شده حاکی از توانایی بالای جذب کاتیون‌هایPb2+، Cd2+، Ni2+ و Zn2+ توسط سیلیکا آئروژل اصلاح سطح شده با آمینو پروپیل تری اتوکسی سیلان بود؛ به طوری که می‌توان از آن در تصفیه آب و فاضلاب‌های صنعتی و شهری استفاده کرد.

Title: برآورد میزان فعاليت واحدهای صنعتی در زمینه بازیافت در منطقه

http://hsr.mui.ac.ir/index.php/jhsr/article/view/105

مقدمه: مقوله اقتصاد، حوزه متنفذي است که تعيين کننده پتانسيل فعاليت در هر کاري است. مطالعه بازار يکي از روش‌هاي مؤثر در ارزيابي اقتصادي است. هدف این مطالعه، تعيين ميزان جذابيت‌هاي سرمايه‌گذاري و کاري در حوزه بازیافت بازار کنوني بازيافت در سطح استان اصفهان و تعيين شاخص فعاليت آن‌ها است، که مورد بررسی و مطالعه قرار گرفت. روش‌ها: ابتدا مواد بازيافتني دسته‌بندي شدند و سپس اطلاعات تمامي واحدهاي صنعتي فعال در زمينه تبديل مواد بازيافتني مستقر در شهرک‌هاي صنعتي و خارج از شهرک‌ها مورد بررسي قرار گرفتند. در اين مقاله از روش مطالعات کمی و پژوهش‌های تجربی بهره گرفته شد. در هر کدام از حوزه‌هاي بازيافتي ميزان اشتغال، سرمايه‌گذاري و ظرفيت توليد واحدهاي صنعتي تعیین شدند و شاخص فعاليت هر کدام بر اساس نتايج به دست آمده مشخص گرديد. یافته‌ها: در سطح استان بیشترین شاخص فعاليت مربوط به دسته فلزات و بعد کاغذ و کارتن می‌باشد و در زمینه بازیافت زایدات الکترونیکی و خودرو فرسوده کاری صورت نگرفته است. نتیجه‌گیری: از این مطالعات می‌توان در جهت برنامه‌ریزی جهت توسعه صنعت بازیافت در سطح استان استفاده کرد.

Title: بررسی پهنه‌بندی پسماند صنعتی و شهری در سطح استان اصفهان

http://hsr.mui.ac.ir/index.php/jhsr/article/view/106

مقدمه: توليد مواد زايد جامد، محصول فعاليت‌هاي مختلف انسان است كه امروزه با تغيير شيوه زندگي و توسعه همه جانبه نسبت به گذشته، تغييرات زيادي داشته است. در استان اصفهان روزانه پسماند زيادی توليد می‌گردد. با توجه به اهميت پسماند و بازيافت آن‌ها، هدف تعيين محدوده‌های توليد پسماندهای مختلف جهت مکان‌يابی مراکز مناسب برای بازيافت پسماندها است. روش‌ها: جهت بررسی پهنه‌بندی پسماند صنعتی و شهری در سطح استان اصفهان، وضعيت توليد پسماند گروه‌های مختلف در سطح استان به صورت کمی و کيفی مطالعه و برآورد گرديد. با توجه به نوع صنايع استان، پسماندها را می‌توان به ده دسته فلز، شيشه، زايدات ساختماني و سنگ‌بري‌ها، لاستيک و پلاستيک، کاغذ و مقوا، زايدات الکترونيک، خودرو فرسوده، منسوجات، چوب و ساير تقسيم کرد. در اين مقاله نقشه‌هاي تلفيقی پراکندگي پسماند توليدي گروه‌هاي مختلف در شهرک‌هاي صنعتي، صنايع پراکنده و شهرستان‌ها توسط نرم‌افزار Arc GIS رسم گرديد، تا بر اساس توزيع اين پسماندها، سازمان‌های مربوطه مکان مناسب برای بازيافت اين پسماندها را در نظر گيرند. یافته‌ها: در نقشه‌هاي تهيه شده، توزيع مواد بازيافتني کاني غير فلزي (شيشه)، سلولزي (چوب)، شيميايي (لاستيک و پلاستيک) و منسوجات، زایدات الکترونيکي و الکتريکي و خودرو فرسوده نشان داده شده است، که با توجه به تجمع آن‌ها مکان‌های مناسب جهت بازيافت پسماندهای مختلف قابل تشخيص است. نتیجه‌گیری:

Title: بررسي كارايي سيستم رآکتور بی هوازی بیوفیلمی پر و خالی شونده بطور متوالی

http://hsr.mui.ac.ir/index.php/jhsr/article/view/121

مقدمه: دفع پساب كارخانه شكر به رودخانه‌ها و محيط زيست موجب آلودگي و به خطر افتادن حيات آبزيان و محيط زيست مي‌شود، لذا تصفيه پساب اين كارخانه امري ضروري تلقي مي‌شود. هدف از اين پژوهش، سنجش کاهش بار آلودگي صنایع نیشکر با استفاده از راكتور بيوفيلم بي‌هوازي پر و خالي شونده به طور متوالي (ASBBR) است. روش‌ها: در اين پژوهش راكتور ASBBR جهت تصفيه پساب كارخانه نيشكر اميركبير به مدت 8 ماه مورد استفاده قرار گرفت. حجم كلي راكتور 7 ليتر بود. اين راكتور با بارگذاري حجمیgCOD/L.d  25/0 و بارگذاری سطحی  mgCOD/m2.d 33/ با استفاده از ملاس كارخانه به‌عنوان سوبستره اصلي با COD و BOD5 به‌ ترتيب 1075 و 450 ميلي‌گرم در ليتر در دماي ºC35 راه‌اندازي شد. رژيم بهره‌برداري از راكتور شامل 23 دقيقه زمان تغذيه، 22 ساعت و 14 دقيقه زمان واكنش، 1 ساعت زمان ته‌نشيني و 23 دقيقه زمان تخليه بود. يافته‌ها: بارگذاري حجمی و سطحی بهینه به ‌ترتیبgCOD/L.d  8 و mgCOD/m2.d 67/10 بود كه در اين بارگذاري‌ها COD به ‌طور متوسط از mg/l 351 ± 32270 به mg/l 2222 ± 6146 با راندمان 7 ± 9/80 درصد كاهش پيدا كرد و بيشترين راندمان حذف سیستم در این بارگذاری برابر با 5/91 % و ميزان بيوگاز توليدی 74/3 لیتر به ازای هر لیتر حجم راکتور به‌ دست آمد. نتيجه‌گيري: این سیستم قادر به حذف مقادیر بالای COD تا حدود  mg/l32000 می‌باشد. بنابراين راكتور گزينه مناسبي جهت تصفيه پساب كارخانه‌هاي توليد شكر مي‌باشد.

Title: کاربرد نانو‌ذرات اکسید نیکل به عنوان یک جاذب کارآمد برای حذف رنگ از پساب‌های سنتتیک

http://hsr.mui.ac.ir/index.php/jhsr/article/view/55

مقدمه:در این تحقیق کارایی فرایند جذب با استفاده از نانو‌ذرات اکسید نیکل برای تصفیه پساب حاوی رنگ مونوآزوی نارنجی 2 (Orange II) در مقیاس آزمایشگاهی مورد مطالعه قرار گرفت. روش‌ها‌: تأثیر عوامل مختلف از جمله مقدار جاذب، زمان تماس، pH و غلظت اولیه رنگ‌زا بررسی شد. یافته‌ها: مناسب‌ترین محدوده pH برای رنگ‌زا، pH اسیدی می‌باشد و تحت این شرایط، می‌توان mg/L 50 رنگ‌زای مورد نظر را با g/L 6/0 اکسید نیکل به طور کامل حذف نمود. این غلظت به عنوان غلظت بهینه اکسید نیکل تعیین گردید. با این که افزایش غلظت رنگ‌زا موجب کاهش بازدهی فرایند شد، ولی کارایی قابل توجهی تا غلظت mg/L 100 رنگ‌زا به دست آمد. نتیجه‌گیری: داده‌ها نشان دادند که نانوذرات اکسید نیکل می‌تواند به عنوان یک جاذب کارآمد برای رنگ‌بری رنگ‌زاهای آزو مورد استفاده قرار گیرد.

Title: تأثير وضعيت تحصيلي مديران بر شاخص‌هاي بهداشتي مراکز خدمت‌رسان به زائران

http://hsr.mui.ac.ir/index.php/jhsr/article/view/124

مقدمه: از عوامل اصلي انتخاب مکان اقامت واعتماد مسافران در سفرهاي زيارتي و گردشگري بهداشت هتل‌ها است. شهرقم، با حدود 17 ميليون نفر زائر در سال، دومين مرکز زيارتي کشور، از لحاظ تردد گردشگر مي‌باشد. اين تحقيق به منظور بررسي ميزان تأثير مدرک تحصيلي مديران داخلي و مسؤولين فني هتل‌ها و اماکن زيارتي شهر قم بر وضعيت شاخص‌هاي بهداشت محيط اين مراکز انجام شد. روش‌ها: براي تعيين شاخص‌هاي بهداشت محيطي اين اماکن، پرسشنامه‌اي مشتمل بردو بخش دموگرافيك و بخش اختصاصي بر اساس آيين‌نامه بهداشت محيط اماکن عمومي، مصوب وزارت بهداشت، درمان و آموزش پزشکي تنظيم گرديد. تكميل پرسشنامه از طريق مصاحبه حضوري و بازديد از كليه اماكن اقامتي داراي مجوز سازمان ايرانگردي و جهانگردي شهر قم شامل49 امکنه طي ماه آذر لغايت بهمن ماه، سال 85 انجام شد. يافته‌ها: تعداد مديران داخلي داراي مدرك تحصيلي زير ديپلم ‌24 نفر و مديران داراي مدرك تحصيلي ديپلم، ليسانس، فوق‌ليسانس و بالاتر به ‌ترتيب 13، 4، 9 و 2 نفر گزارش شد.‌ با استفاده از آزمون T و اطمينان 95 درصد، تفاوت امتياز وضعيت بهداشتي در مراکز اقامتي خدمت‌رسان به زائرين در گروه زير‌ديپلم و بالاي ديپلم معني‌دار بود (p=0.01). نتيجه‌گيري: با توجه به تأثيرات مثبت به کارگيري افراد تحصيل‌ کرده به عنوان مديران داخلي و مسؤولين فني مراکز اقامتي خدمـت‌ر‌سان به زائران، توصيه مي‌شود تمهيدات قانوني به منظور التزام متصديان مراکز اقامتي جهت استفاده از افراد داراي مدارک تحصيلي دانشگاهي در نظر گرفته ‌‌شود.

Title: اندازه‌گیری غلظت رادیونوکلئید مصنوعی در نمونه‌های آب و رسوبات رودخانه اروندرود

http://hsr.mui.ac.ir/index.php/jhsr/article/view/139

مقدمه: با توجه به این که تخليه بعضی از پساب‌های صنعتی به رودخانه اروندرود صورت می‌گیرد، احتمال حضور مواد رادیو‌نوکلئوتید در رودخانه وجود دارد. در اين مطالعه هدف بررسي میزان پرتوزایی زمین منطقه اروندرود تا دهانه خليج فارس بوده است. روش‌ها: غلظت راديو‌نوکلئيد سزیم -137 (Cs137) در 20 نمونه رسوب و 8 نمونه آب نمونه‌برداري شده به ترتیب از20 و 8 ايستگاه مختلف در رودخانه اروند اندازه‌گيري شد. تعيين ميزان پرتوزايی راديونوكلئيد‌هاي ذكرشده در نمونه‌هاي رسوب به روش گاما اسپکترومتری انجام پذيرفته است. تجزیه آماری داده‌ها بااستفاده از نرم افزار spss و روش مقایسه میانگین یک طرفه (on-way anova) و مقایسه میانگین‌ها به روش دانکن صورت گرفت. یافته‌ها: ميزان پرتوزايي راديونوكلئيد مصنوعي سزیم 137 در نمونه‌هاي رسوب در گستره 1.13 تا 4.16 Bq.kg-1 ب دست آمده‌است. نتيجه‌گيري: غلظت‌هاي به دست آمده در نمونه‌های آب کمتر از حد (Minimum Detectable Activity) MDA سیستم شمارش بوده‌اند به طوری که استفاده از آب رودخانه به عنوان آب شرب ، از نظر این رادیونوکلئید تهدیدی برای سلامت ساکنان منطقه ایجاد نمی‌کند.

Title: تأثیر بازدارندگی آنتی‌بیوتیک‌های آمپی‌سیلین و جنتامایسین بر میزان متان‌سازی بیومس بی‌هوازی

http://hsr.mui.ac.ir/index.php/jhsr/article/view/47

مقدمه: درصد بالایی از آنتي‌بيوتيك‌ها که توسط انسان‌ها و حیوان‌ها مصرف می‌‌شوند پس از تأثیر‌گذاری همراه سایر ضایعات از بدن دفع می‌شوند. این ترکیبات با ورود به فاضلاب، می‌توانند فرايند تصفيه بی‌هوازی را مختل کنند. در اين مطالعه، رفتار بازدارندگی دو آنتي‌بيوتيك آمپی‌سیلین و جنتامایسین بر فعاليت متان‌سازي ويژه بيومس بي‌هوازي (SMA: Specific Methanogenic Activity) مورد بررسي قرار گرفت. روش‌ها: 18 آزمون به روش ناپيوسته در ويال‌هاي شيشه‌اي  mL120 صورت گرفتند که 37 درصد از حجم هر ویال به بیومس، 40 درصد سوبستره و 23 درصد به تجمع بیوگار اختصاص یافت. هر تست 25ـ20 روز به طول انجاميد. متان توليد شده به وسيله جايگزيني گاز با محلول 2 نرمال KOH به عنوان جاذب  CO2اندازه‌گيري شد. اسید‌های چرب مانند استیک، پروپیونیک، بوتیریک اسید به عنوان سوبستره کمکی استفاده گردید. يافته‌ها: در این مطالعه در غلظت‌های 200، 500 و mg/L 1000 آمپی‌سیلین، متان‌سازی ویژه تجمعی به ترتیب 25، 35 و 46 برای پروپیونیک، 66، 101 و 154 برای استیک و 198، 140 و mL/g VSS. d 245 برای بوتیریک به دست آمد. همچنین در غلظت‌های 100، 500 و mg/L 1000 جنتامایسین تولید متان‌سازی ویژه تجمعی به ترتیب 54، 72 و 71 برای پروپیونیک، 141، 204 و 257 برای استیک، 139، 74 و mL/g VSS. d 5/85 برای بوتیریک به دست آمد. نتيجه‌گيري: آمپی‌سیلین در غلظت‌های مشابه نسبت به جنتامایسین اثر بازدارندگی بیشتری بر بیومس بی‌هوازی دارد. همچنین، پروپیونیک نسبت به استیک و بوتیریک اسید بازدارنده‌تر است.

Title: بررسی کیفیت شیمیایی آب‌های زیر‌زمینی در مجاورت محل دفن زباله شهر یزد در سال ١٣٨8

http://hsr.mui.ac.ir/index.php/jhsr/article/view/49

مقدمه: امروزه آب زیرزمینی مهم‌ترین منبع تأمین نیازهای آبی بخش کشاورزی و صنعت محسوب می‌شود. از این رو نیاز به حفاظت این منبع ارزشمند در مقابل آلودگی‌ها، بیش از گذشته احساس می‌شود. در این مطالعه، اثرات احتمالی محل دفن زباله شهر یزد به عنوان یک منبع بالقوه آلودگی بر آب‌های زیرزمینی منطقه، مورد بررسی قرار گرفت. روش‌ها: تعداد دو حلقه چاه در پایین‌دست و یک حلقه چاه شاهد در بالا‌دست محل دفن زباله انتخاب شده، مورد نمونه‌برداری قرار گرفتند. نمونه‌ها از نظر پارامترهای pH، هدایت الکتریکی (EC)، کلرور (Cl-)، نیترات (-3NO)، سختی کل، قلیاییت و فلزات سنگین شامل سرب (Pb)، کادمیوم (Cd)، مس (Cu) و روی (Zn) آزمایش شدند. آنالیز آماری داده‌ها با استفاده از آزمون t-test و ترسیم نمودارها با نرم‌افزار Excel انجام گرفت. یافته‌ها: pH آب پایین‌دست به نحو معنی‌داری کمتر از pH آب بالادست مشاهده شد (038/0 = P). همچنین مقادیر پارامترهای هدایت الکتریکی، کلرور، نیترات، سختی و قلیاییت در آب پایین‌دست افزایش معنی‌داری نشان داد (05/0 < P)، اما افزایشی در غلظت فلزات سنگین در ایستگاه‌های پایین‌دست مشاهده نگردید. نتیجه‌گیری: نتایج مطالعه نشان داد که با افزایش فاصله از محل دفن، کیفیت شیمیایی آب زیرزمینی بهبود می‌یابد. بنابراین بهتر است که برداشت آب در فواصل نزدیک به محل دفن انجام نشود. البته اصولی‌ترین اقدام، عایق‌کشی کف محل دفن زباله به منظور کنترل حرکت آلاینده‌های موجود در شیرابه می‌باشد.

Title: مواد زائد الکترونیکی، چالشی نوظهور در عرصه مدیریت بهداشت محیط کشور

http://hsr.mui.ac.ir/index.php/jhsr/article/view/184

تولید زائدات الکترونیکی به عنوان بخشی از زائدات جامد تولیدی در جوامع امروزی به سرعت در حال رشد است. این زائدات 5-2 درصد زائدات جامد شهری را تشکیل می‌دهند و انواع پسماندهای الکترونیکی را شامل می‌شوند. در خاور میانه، ایران بزرگ‌ترین بازار مصرف محصولات الکترونیکی را دارد و متعاقب آن بیشترین مواد زائد جامد الکترونیکی نیز متعلق به ایران است. به طور تقریبی هر سال حدود یک میلیون و 200 هزار کامپیوتر در ایران مونتاژ شده است. بیش از 40 مییون تلفن همراه در ایران فعال است. تولید زائدات الکترونیکی در ایران بیش از 37000 تن در سال برآورد می‌شود. سازمان‌های دولتی و خصوصی برای تأمین نیاز‌های دولت ایران در اجرای مدیریت زائدات باید با یکدیگر همکاری کنند. قانون‌گذاری می‌تواند مدیریت زائدات الکترونیکی را بهبود بخشد. امروزه بسیاری از کشورها به ویژه کشورهای پیشرفته، به تدوین قوانین سخت‌گیرانه برای مدیریت زائدات الکترونیکی می‌پردازند.
